# Investigation of Skin Circulation Hemodynamics Using Skin Laser Speckle Flowgraphy After Trapezius Muscle Self-Stretching

**DOI:** 10.3390/muscles5020031

**Published:** 2026-04-30

**Authors:** Miki Yoshimura, Takanori Taniguchi, Takeshi Yoshitomi, Yuki Hashimoto

**Affiliations:** 1 Department of Orthoptics and Visual Sciences, Faculty of Health and Medical Sciences, Graduate School of Health and Welfare Sciences, International University of Health and Welfare, Akasaka 4-1-26, Minato-ku, Tokyo 107-8402, Japan; yoshimura.m08@gmail.com (M.Y.); yoshitomi@ihwg.jp (T.Y.); 2 Department of Physical Therapy, Faculty of Medicine, Fukuoka International University of Health and Welfare, Momochihama 3-6-40, Sawara-ku, Fukuoka 814-0001, Japan; taniguchi.tg@ihwg.jp; 3 Department of Orthoptics, Faculty of Medicine, Fukuoka International University of Health and Welfare, Momochihama 3-6-40, Sawara-ku, Fukuoka 814-0001, Japan

**Keywords:** laser speckle flowgraphy, mean blur rate, self-stretching, ultrasound strain elastography, upper trapezius muscle

## Abstract

Stretching of the upper trapezius muscle reduces stiffness and choroidal blood flow velocity, but its effect on skin blood flow remains unclear. We evaluated the changes in upper trapezius skin circulation hemodynamics before/after self-stretching using skin laser speckle flowgraphy (LSFG). Twenty-two healthy young adults (median age [Q1–Q3]: 21.0 [20.0–21.0] years) were enrolled. Trapezius stiffness was assessed using ultrasound strain elastography, and skin and choroidal blood were measured with skin and ocular LSFG, respectively, using mean blur rate (MBR) as an index of blood flow velocity. Intraocular pressure (IOP); systolic (SBP), diastolic (DBP), and mean blood pressure (MBP); heart rate (HR); ocular perfusion pressure (OPP); salivary α-amylase (sAA) activity; and subjective eyestrain/shoulder stiffness symptoms (visual analog scale, VAS) were evaluated at baseline and after stretching. SBP, DBP, MBP, OPP, sAA activity, VAS scores for eyestrain and shoulder stiffness, trapezius stiffness, and skin and choroidal MBR decreased significantly after self-stretching, whereas IOP and HR remained unchanged. Trapezius muscle self-stretching reduces muscle stiffness and induces relaxation in healthy adults, accompanied by reduced sympathetic activity and decreased systemic, choroidal, and local skin circulation. These findings suggest that skin LSFG may serve as a useful, non-invasive tool for evaluating shoulder stiffness.

## 1. Introduction

Shoulder stiffness is a prevalent musculoskeletal system disorder, with a lifetime prevalence of approximately 30–70% [1,2]. In recent years, the number of individuals experiencing shoulder stiffness has been increasing, likely due to prolonged use of computers and smartphones [2,3,4]. Shoulder stiffness adversely affects daily life, academic performance, and mental health [5,6]. Among office workers, it has also been found to reduce work productivity and quality of life, making it a significant occupational health issue [7].

Shoulder stiffness is primarily related to stiffness of the trapezius muscle [8,9]. The trapezius is a superficial muscle extending from the neck to the shoulders and upper back, playing an important role in maintaining cervical and shoulder girdle posture as well as facilitating movements of the head and shoulder. Trapezius muscle stiffness has been reported to affect not only neck and shoulder pain but also autonomic nervous system activity [10]. Therefore, appropriate assessment and management of trapezius muscle stiffness in individuals with shoulder stiffness are critically important.

Recent studies have increasingly utilized ultrasonography equipped with elastography to measure muscle elastic modulus as an index of muscle stiffness [11,12]. Elastography enables quantitative assessment of muscle elastic properties through measurements such as strain ratio and shear-wave velocity [13,14], making it a method of growing interest for direct and precise evaluation of trapezius muscle stiffness.

Various interventions, including thermotherapy [8], ultrasound therapy [9], and self-stretching [15,16,17], have been reported to improve trapezius muscle stiffness. Among these, self-stretching is a highly practical intervention that requires no specialized equipment or therapists and can be performed anytime and anywhere, contributing to its widespread use. In addition, visually demanding work such as computer use can cause eye discomfort and neck/shoulder strain [18]. Symptoms may include dry eyes, blurry vision, discomfort, and pain, and it has been suggested that these are related to abnormalities in the anterior segment and extraocular muscles, including decreased blinking, tearing, pupillary reflexes, and accommodative or vergence ability [19,20,21,22]. In contrast, cumulative working hours, astigmatism, accommodative response, and concomitant ocular discomfort have been found to exacerbate neck and shoulder discomfort, while no significant effect was observed on extraocular discomfort such as burning or smarting. An interaction effect has also been reported: participants with greater increases in internal eye discomfort developed more neck and shoulder discomfort over time [18].

The posterior segment of the eye includes the retina and choroid, both of which contain distributed blood vessels. Autoregulation varies across vascular systems, but retinal vessels exhibit stronger autoregulatory capacity than choroidal vessels [23]. Changes in ocular perfusion pressure (OPP) immediately after dynamic exercise have been shown to increase retinal–choroidal blood flow, with the increase more sustained in the choroid than in the retina. This difference may reflect stronger autoregulatory mechanisms in the retina compared to the choroid [24]. The choroid, which accounts for about 90% of ocular blood flow, supplies oxygen and nutrients to photoreceptors and is strongly affected by the autonomic nervous system due to its limited autoregulatory capacity [25,26]. Recent evidence suggests that choroidal circulation in the posterior fundus may be associated with shoulder stiffness and eyestrain [17].

Laser speckle flowgraphy (LSFG) provides reproducible, non-invasive data on choroidal circulation dynamics in both diseased and normal eyes [17,27,28,29,30,31]. For example, in central serous chorioretinopathy—where sympathetic hyperactivity due to stress-prone personality and hypertension contributes to pathogenesis [27]—and in hypertensive chorioretinopathy [28], choroidal circulation increases during the acute phase and decreases during remission. Cold pressor tests in healthy individuals have demonstrated that temporary stress enhances sympathetic dominance, increasing systemic and choroidal circulation while reducing choroidal thickness [29]. Conversely, heat therapy induces parasympathetic dominance, reducing systemic circulation and consequently lowering choroidal circulation [30,31]. LSFG thus enables sensitive detection of autonomic nervous system changes. Moreover, studies showing significant increases in choroidal blood flow velocity with rising intraocular pressure (IOP), blood pressure (BP), and OPP after isometric exercise in healthy individuals indicate that choroidal circulatory dynamics depend on systemic circulatory dynamics [24,32,33].

Self-stretching of the trapezius muscle may enhance parasympathetic nervous system activity [34,35]. In healthy adults in their 20s, short-term trapezius self-stretching has been shown to reduce muscle elastic modulus and enhance parasympathetic activity, potentially leading to decreased systemic and choroidal circulation parameters [17]. In contrast, in participants in their 40s, only the elastic modulus showed no significant change after self-stretching, suggesting that age influences the effects of short-duration stretching [17]. However, no studies have investigated changes in skin blood flow velocity in the upper trapezius muscle after self-stretching in healthy adults.

Skin-LSFG is a laser-based imaging method that enables visualization of microcirculatory dynamics in two-dimensional images [36,37,38,39]. By irradiating near-infrared laser light onto the skin surface and capturing the reflected signal, it generates color-coded images similar to thermography while allowing quantification. The short measurement time minimizes subject burden. The measurement range involves irradiating the skin surface and observing depths of approximately 1–2 mm into the dermis, which contains numerous blood vessels. Limitations include sites prone to movement during measurement (e.g., due to respiration or tremors), areas with pigmentation, and irregular skin surfaces, which complicate measurement. Thus, measurement is best suited for sites where fixation is possible. Because values are relative rather than absolute, comparisons are possible within the same site and subject. Previous studies have used this technique to measure skin blood flow velocity before and after facial self-massage [37], to evaluate perfusion in breast reconstruction surgery [38], and to assess lower limb blood flow in patients with severe ischemia after revascularization [39]. These findings suggest that skin blood flow velocity can be measured in various body regions.

Therefore, we hypothesize that self-stretching for shoulder stiffness reduces trapezius muscle elasticity, promotes parasympathetic dominance, and decreases systemic and choroidal circulation, along with reduced skin circulation dynamics in the trapezius. This may indicate that self-stretching alters not only trapezius muscle elasticity but also skin circulation, including systemic and choroidal dynamics. However, the effect of trapezius self-stretching on skin blood flow has not yet been evaluated. In this context, the present study aimed to investigate the skin circulatory dynamics of the upper trapezius muscle before and after self-stretching using skin LSFG in healthy young adults.

## 2. Results

This study included 22 healthy adult volunteers (14 females and 8 males). The median age was 21.0 [20.0–21.0] years (range, 18–39 years). The median refractive error was −2.38 [−4.38–−0.59] D (range, +0.50 D to −11.50 D). All volunteers demonstrated best-corrected visual acuity (BCVA) of 20/20 or better.

### 2.1. IOP, BP, HR, OPP, sAA Activity and VAS

Changes in IOP, systolic blood pressure (SBP), diastolic blood pressure (DBP), mean blood pressure (MBP), heart rate (HR), OPP, salivary α-amylase (sAA) activity, and visual analog scale (VAS) scores are shown in Table 1. Compared with baseline, SBP (*p* < 0.001), DBP (*p* = 0.003), MBP (*p* < 0.001), and OPP (*p* < 0.001) decreased significantly. No significant changes were observed in IOP (*p* = 0.262) or HR (*p* = 0.354). sAA activity, a biomarker of sympathetic nervous system activation, decreased significantly compared with baseline values (*p* = 0.025), suggesting a shift toward reduced sympathetic dominance. Subjective symptoms of eyestrain and shoulder stiffness, assessed using VAS, were 2.9 [1.8–5.3] cm and 3.6 [0.8–6.5] cm at baseline, and decreased significantly to 1.9 [0.8–3.8] cm and 0.9 [0.2–2.9] cm, respectively, after self-stretching (*p* = 0.017 and *p* < 0.001).

### 2.2. Stiffness of the Upper Trapezius Muscle

**Figure 1:**
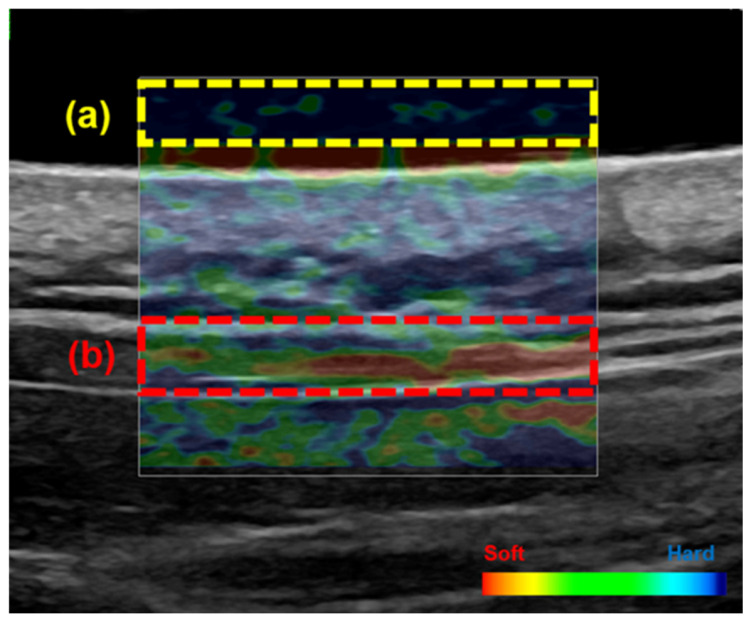
Ultrasound strain elastography image of the upper trapezius muscle. Regions of interest (ROIs) were defined for the acoustic coupler gel (**a**) and the upper trapezius muscle (**b**). Areas displayed in red represent softer tissue, whereas areas shown in blue indicate stiffer tissue. Muscle elasticity was quantified by calculating the strain ratio between the trapezius muscle and a gel pad, using a scale ranging from 0 (softest) to 6 (stiffest). A higher strain ratio indicated greater muscle stiffness.

Changes in upper trapezius muscle hardness are summarized in Table 1. Muscle stiffness was 0.5 [0.4–0.5] at baseline and 0.4 [0.2–0.5] after self-stretching, showing a significant decrease (*p* < 0.001) (Table 1, Figure 1). To evaluate measurement reproducibility by a single examiner, stiffness was measured three times, and the intraclass correlation coefficient (ICC) was calculated. The intra-examiner reliability was high (ICC = 0.77), showing good reproducibility.

### 2.3. Choroidal and Skin MBR

**Figure 2:**
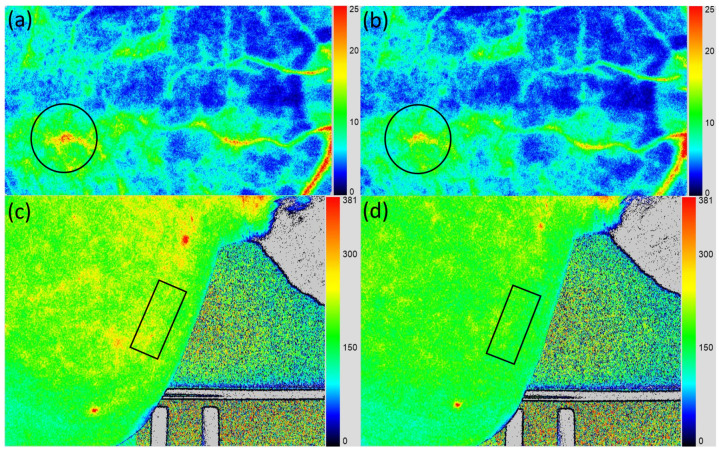
Ocular and skin laser speckle flowgraphy (LSFG). Participants at baseline (**a**,**c**) and after upper trapezius self-stretching (**b**,**d**). The choroidal mean blur rate (MBR) was measured by placing a rubber band (150 × 150 pixels) over the macula. Skin MBR was measured at the midpoint between the C7 spinous process and the acromion, using a 156 × 67 pixels rubber band. Blue indicates low MBR, and red indicates high MBR.

Choroidal mean blur rate (MBR) values are shown in Table 1. The median value was 13.9 [12.1–17.8] at baseline and 12.4 [10.6–15.7] after trapezius self-stretching, representing a significant decrease of −10.8% [5.8–15.3] (*p* < 0.001). Similarly, skin MBR in the upper trapezius muscle was 185.5 [171.1–200.7] at baseline and 169.1 [163.5–176.9] after self-stretching, decreasing significantly by −6.7% [1.1–12.3] (*p* = 0.009) (Table 1, Figure 2).

## 3. Discussion

This study is, to the best of our knowledge, the first study to investigate the skin circulatory hemodynamics of the upper trapezius muscle before and after self-stretching. In the present study, SBP, DBP, MBP, OPP, sAA activity, subjective symptoms of shoulder stiffness and eyestrain, trapezius muscle stiffness, and skin and choroidal blood flow velocity all decreased significantly after trapezius self-stretching in healthy young adults. These findings indicate that trapezius self-stretching affects not only muscle elasticity but also choroidal and skin blood flow velocity. Furthermore, these changes are associated with parasympathetic dominance and a subsequent decrease in BP.

Choroidal vessels exhibit poor autoregulation and are directly influenced by the autonomic nervous system [23,25,26]. Previous studies in healthy young individuals have shown that sympathetic nerve dominance—for example, after a cold pressor test or during winter—increases systemic circulation and elevates choroidal circulation as measured by ocular LSFG [29,40]. In contrast, parasympathetic dominance—for example, after warm water immersion or periocular skin warming—reduces systemic and choroidal circulation dynamics [30,31]. These changes in choroidal circulation and morphology are linked to alterations in cardiac output and vascular function due to autonomic activity. Sympathetic activation increases cardiac output and choroidal blood flow [28,29,41], while also causing vasoconstriction of choroidal arteries and reducing choroidal thickness [40,42,43]. Conversely, parasympathetic activation induces choroidal vasodilation via cholinergic and nitric oxide (NO)-mediated mechanisms [44,45], while simultaneously reducing cardiac output through inhibitory effects on heart rate and cardiac function [44,45]. Together, the combination of reduced driving pressure and vasodilation may lead to decreased choroidal blood flow velocity. Unlike ischemic reductions in blood flow, which are pathological, the parasympathetic-mediated decrease observed here reflects a physiological relaxation response.

The results of this study support the effectiveness of static stretching in reducing trapezius muscle tension. Static stretching has been reported to reduce muscle stiffness by altering the viscoelastic properties of the muscle–tendon unit [46]. Specifically, increased flexibility in elastic components reduces resistance to applied force, potentially improving range of motion. Enhanced local blood flow through stretching also promotes oxygen delivery and removal of metabolic byproducts, further reducing muscle tension [47]. In addition to these physical effects, trapezius stretching influences the autonomic nervous system by promoting parasympathetic activity, reducing heart rate variability and muscle tension, and producing a relaxing effect [34,35]. This may contribute to improvements in shoulder stiffness and stress-related muscle tension. A recent report showed that choroidal blood flow velocity, SBP, DBP, MBP, and sAA activity decreased significantly after trapezius self-stretching in both 20- and 40-year-old groups. However, trapezius muscle stiffness decreased significantly only in the younger group, with no measurable change in the older group [17]. Thus, age may attenuate the effects of short-term self-stretching on trapezius stiffness [8].

Skin LSFG can detect subtle changes in skin blood flow that thermography cannot capture. This suggests its potential usefulness for evaluating skin blood flow in ischemic wound assessment [36]. Previous studies using skin LSFG have reported increased skin blood flow after cosmetic roller massage [37], objective evaluation of perfusion in breast reconstruction surgery [38], and improved peripheral circulation after revascularization in patients with arterial disease [39]. In the present study, the significant decrease in trapezius skin blood flow velocity after stretching demonstrates that skin LSFG is also useful for assessing circulation dynamics in the shoulder.

The effects of trapezius self-stretching may involve not only physiological mechanisms but also molecular signaling pathways [48,49]. In particular, the mammalian target of rapamycin (mTOR) signaling pathway may play a role beyond muscle metabolism, potentially influencing autonomic activity and vascular dynamics. Mechanical stretching of muscle cells induces plasma membrane tension fluctuations, activating mTORC1 signaling, which converts mechanical stimuli into molecular responses associated with muscle adaptation and tissue remodeling [50]. Moreover, mTORC1 signaling in the hypothalamus has been shown to regulate sympathetic activity and arterial blood pressure [51]. These findings suggest that mTOR may contribute to circulatory regulation via central nervous system pathways. Taken together, reductions in muscle tension and changes in choroidal and skin blood flow velocity observed after trapezius stretching are likely driven primarily by immediate autonomic regulation, with molecular signaling pathways potentially contributing as underlying mechanisms. However, because mTOR activity was not directly assessed in this study, these interpretations remain speculative, and more research is needed to clarify the role of mTOR-related signaling in the observed effects.

The findings of this study suggest several potential clinical implications. First, self-stretching of the upper trapezius muscle was associated with reductions in local muscle stiffness and changes in systemic and regional circulatory dynamics, including choroidal and cutaneous blood flow velocity, accompanied by decreases in blood pressure and indices consistent with sympathetic activity. These preliminary results indicate that a simple, low-cost, and non-invasive intervention such as self-stretching may exert broader physiological effects beyond the musculoskeletal system. Second, the observed shift toward parasympathetic dominance following trapezius muscle stretching raises the possibility of benefits for individuals experiencing stress-related symptoms, such as shoulder stiffness and eyestrain, which are common in modern sedentary lifestyles. From a clinical perspective, incorporating trapezius self-stretching into daily routines or rehabilitation programs may contribute to autonomic regulation and stress reduction, although further longitudinal studies are required to confirm these effects. Third, the ability of LSFG to detect subtle changes in skin blood flow in the shoulder region highlights its potential utility for evaluating local circulatory responses to therapeutic interventions. Taken together, these findings underscore the potential clinical relevance of upper trapezius self-stretching as a multifaceted intervention that warrants further investigation in preventive care, occupational health, and rehabilitation contexts, as well as in populations with autonomic dysfunction or circulatory disturbances.

This study has certain limitations. First, the sample size was small and limited to healthy young adults, and the study design was non-randomized without a control group, which limits causal inference and generalizability. Previous studies have reported that short-duration stretching does not alter trapezius muscle stiffness in middle-aged individuals [17], and the effects in elderly populations remain unclear. Second, only the short-term effects of self-stretching were examined. Third, choroidal evaluation was restricted to LSFG. Because MBR is a relative indicator of blood flow velocity, it is not suitable for direct inter-subject comparisons. Furthermore, baseline variability of MBR measurements across long-term sessions and within individuals has not yet been established. Future studies should expand the sample size to include older adults and individuals with chronic shoulder stiffness or eyestrain, employ randomized controlled designs, and investigate the long-term effects of self-stretching. Exploration of other therapies, as well as combined interventions incorporating stretching with different treatments, would also be valuable. Moreover, multimodal evaluation of choroidal morphology and circulation using enhanced depth imaging optical coherence tomography (OCT), swept-source OCT, and OCT angiography, in addition to LSFG, would provide a more comprehensive understanding. Finally, while exploratory, these findings raise the possibility that trapezius self-stretching could be further evaluated in relation to preventing or improving symptoms of systemic diseases involving autonomic dysfunction and blood pressure regulation, as well as conditions such as central serous chorioretinopathy and hypertensive chorioretinopathy.

## 4. Materials and Methods

### 4.1. Participants

This study was approved by the Ethics Committee of Fukuoka International University of Health and Welfare (approval ID: 21-fiuhw-003; date of approval: 22 December 2021) and the study was implemented in accordance with the tenets of the Declaration of Helsinki. Written informed consent was obtained from all individuals. This prospective study included 22 healthy adult participants aged 18–39 years (median age, 21.0 years). Post hoc tests confirmed that the sample size was sufficient, yielding a statistical power of 88% (1 − β = 0.88). None of the participants had ophthalmic or systemic diseases. Each underwent BCVA testing, fundus photography, IOP measurement, BP and HR monitoring, autonomic nervous system assessment, evaluation of subjective symptoms of shoulder stiffness and eyestrain, strain elastography, and ocular and skin LSFG.

### 4.2. Self-Stretching of the Trapezius Muscle

The self-stretching program was based on previously reported protocols [15,17] and consisted of seven exercises designed to improve flexibility and mobility of the neck and shoulder girdle, thereby alleviating shoulder stiffness and neck muscle tension. Specifically, the program emphasized static stretching of the cervical muscles and mobility around the scapula.

**Figure 3:**
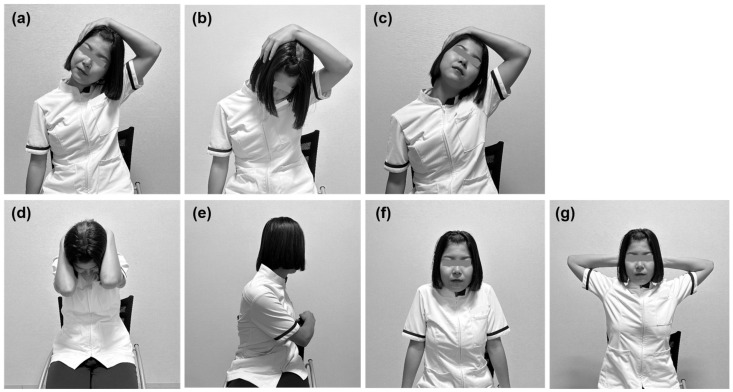
Self-exercises for the trapezius muscle: (**a**) cervical lateral flexion; (**b**) cervical flexion combined with lateral flexion and rotation toward the same side; (**c**) cervical extension combined with lateral flexion and rotation toward the opposite side; (**d**) cervical flexion; (**e**) trunk rotation performed simultaneously with rotation of the neck to the same side; (**f**) scapular elevation and depression; and (**g**) scapular abduction and adduction.

The exercise program consisted of the following movements: (a) cervical lateral flexion; (b) cervical flexion combined with lateral flexion and rotation toward the same side; (c) cervical extension combined with lateral flexion and rotation toward the opposite side; (d) cervical flexion; (e) trunk rotation performed simultaneously with rotation of the neck to the same side; (f) scapular elevation and depression; and (g) scapular abduction and adduction (Figure 3).

Exercises (a)–(e) were performed in five sets of 20 s each, whereas (f) and (g) were performed in three sets of 20 repetitions each. The total duration of the program was approximately 15 min. Each movement was performed slowly, within the range of pain and discomfort. Examinations were conducted in a quiet room at 24 ± 1 °C with humidity maintained at 47 ± 3%. Participants were asked not to smoke or exercise for at least 2 h before the examinations and rested for 10 min in a quiet room prior to testing.

Exercises (a)–(e) were performed in five sets of 20 s each, whereas (f) and (g) were performed in three sets of 20 repetitions each. The total duration of the stretching program was approximately 15 min. Each movement was performed at a slow pace, and participants were encouraged to perform it within the range of pain and discomfort.

### 4.3. IOP and Systemic Hemodynamics

Each participant’s IOP, SBP, DBP, and HR were assessed before and after trapezius self-stretching. IOP was measured using a non-contact tonometer (NT-530, NIDEK Co., Ltd., Aichi, Japan). BP was measured using a digital automatic BP monitor (Omron, OMRON DALIAN Co., Ltd., Kyoto, Japan).

MBP was calculated based on SBP and DBP (1), and OPP was calculated from the MBP and IOP (2).

MBP = DBP + 1/3(SBP − DBP)(1)

OPP = 2/3MBP − IOP(2)

Human and animal studies have shown some degree of autoregulation of choroidal vessels in response to changes in OPP [32,52]. Regulatory mechanisms of choroidal circulation compensate better for increases in BP than for increases in IOP, and for the same OPP, blood flow is better regulated at lower, rather than at higher, IOPs [32]. However, because the choroid is mainly controlled by sympathetic innervation [23,24,26], choroidal autoregulation is also affected by IOP, BP, OPP, and sympathetic input [53].

### 4.4. Autonomic Nervous System Assessment

sAA activity, assessed using a salivary amylase monitor (Nipro Co., Ltd., Osaka, Japan), was used as a noninvasive indicator reflecting plasma norepinephrine concentration to evaluate sympathetic nervous system reactivity. Although sAA is a surrogate marker of sympathetic activity and does not provide a complete evaluation of autonomic nervous system function, it provides a practical alternative to direct measures of autonomic activity. For this measurement, saliva collection paper was placed under the tongue for 30 s, and sAA activity was measured [8].

### 4.5. Subjective Symptoms of Shoulder Stiffness and Eye Strain

Subjective symptoms, such as shoulder stiffness and eye strain, were assessed using an original VAS specifically created for this study. Participants recorded their scores on a 10-cm straight line, with the left end representing 0 (no symptoms) and the right end representing 10 (most severe symptoms). The recorded scores were measured using a 10-cm ruler.

### 4.6. Ultrasound Strain Elastography

Upper trapezius stiffness was evaluated using the strain elastography function of an ultrasound system (LOGIQ P9; GE Healthcare, Tokyo, Japan). Participants were positioned prone and instructed to remain relaxed to minimize involuntary contraction. Based on previously described protocols [54], the measurement site was identified as the midpoint between the spinous process of the seventh cervical vertebra (C7) and the acromion using a tape measure. To ensure reproducibility, the site was marked on the skin. An acoustic coupling gel pad (Echo Gel PAD EP-S-10, 100 mm × 100 mm × 10 mm; Yasojima, Kobe, Japan) was placed at the site, and elastography images were acquired using a linear probe. Compression speed and pressure were standardized by monitoring the waveform on the display, and data were recorded only when the waveform stabilized. In the color-coded elastography images, the trapezius muscle and gel pad were clearly identified (Figure 1). A Q-box, located at the center of the muscle thickness, was designated as the region of interest (ROI). Following previous studies [17], muscle elasticity was assessed by computing the strain ratio between the trapezius muscle and a gel pad, using a scale ranging from 0 (softest) to 6 (stiffest). Higher strain ratio values reflected increased muscle stiffness. Each measurement was obtained three consecutive times by the same examiner, and the mean value was used for subsequent analysis.

### 4.7. Ocular-LSFG

Posterior fundus hemodynamics were measured using the LSFG-NAVI system (Softcare Ltd., Fukuoka, Japan). LSFG involves illuminating the fundus with an 830-nm diode laser to detect red blood cells moving through the choroidal vessels [27,28,29,30,31]. Reflected light from moving erythrocytes produces blurring within the speckle pattern; the faster the cells move, the lower the speckle contrast. The MBR, calculated from variations in blurring proportional to the reciprocal of the square of speckle contrast, provides quantitative, repeatable, and reproducible measurements [55,56,57]. Each measurement required approximately 4 s. LSFG was performed three times at baseline and after stretching.

Because MBR originates mainly from the choroid when retinal vessels are excluded from the measurement site, this value reflects choroidal hemodynamics, particularly at the macula within the foveal avascular zone. Using LSFG Analyzer software (v3.0.47; Softcare Ltd., Fukuoka, Japan), the measurement circle was automatically set at the same site as baseline (Figure 2). Changes in MBR, a quantitative index of relative blood flow velocity, were expressed as a percentage of the baseline value (100%).

### 4.8. Skin-LSFG

Skin blood flow in the upper trapezius region was assessed using LSFG-PI-E (Sofcare Co., Ltd., Fukuoka, Japan). Near-infrared laser light (830 nm) was irradiated onto the skin surface, and reflected signals were captured to evaluate blood flow in real time [36,38]. Each measurement required approximately 4 s and allowed non-invasive, repeatable assessment [39]. A rubber band was used to stabilize the measurement site. Participants lay prone during testing, with the camera perpendicular to the skin surface, and focus was adjusted until alignment was achieved. The measurement site was the midpoint between the seventh cervical spinous process (C7) and the acromion, where a rubber band (156 × 67 pixels) was placed. Measurements were performed three times before and after stretching, and average values were used. For follow-up analysis, LSFG Analyzer software (v3.10.0.0; Softcare Ltd., Fukuoka, Japan) automatically set rectangles at the same sites as baseline (Figure 2). MBR changes were expressed as a percentage of the baseline value (100%).

### 4.9. Statistical Analyses

All results are expressed as the median [Q1–Q3]. The Wilcoxon signed-rank test was used to evaluate changes in IOP, SBP, DBP, HR, MBP, OPP, sAA activity, VAS scores, upper trapezius muscle stiffness, choroidal MBR, and skin MBR before and after self-stretching. Statistical significance was set at *p* < 0.05. All statistical analyses were conducted using BellCurve for Excel (v 4.08; Social Survey Research Information Co., Ltd., Tokyo, Japan). A post hoc power analysis was performed using G*Power (version 3.1) based on the Wilcoxon signed-rank test model. The effect size (dz = 0.55) was estimated from the Z value of the Wilcoxon test. With an α level of 0.05 (two-tailed) and a sample size of 22 pairs, the achieved power was 0.88.

## 5. Conclusions

In this exploratory study, trapezius self-stretching was associated with reduced muscle stiffness and physiological changes consistent with parasympathetic activation, including decreases in systemic, choroidal, and local skin circulation in healthy young adults. These findings emphasize the potential of skin LSFG as a promising, non-invasive tool for objectively assessing shoulder stiffness, monitoring treatment efficacy, and assessing autonomic responses. Furthermore, the changes in skin blood flow may serve as physiological markers for evaluating autonomic responses to therapeutic interventions. However, further research is required to assess the long-term effects of trapezius self-stretching and its applicability to populations with chronic neck and shoulder symptoms.

## Figures and Tables

**Table 1:** The effects of biometric parameters and general eye factors before and after stretching the upper trapezius muscle in healthy adult individuals.

	Baseline (Median [Q1–Q3])	After the Stretch (Median [Q1–Q3])	*p* Value (Wilcoxon Signed-Rank Test)
IOP (mmHg)	15.0 [12.1–15.6]	14.0 [12.1–15.8]	0.262
SBP (mmHg)	111.0 [106.9–118.6]	106.0 [100.1–114.5]	<0.001 ***
DBP (mmHg)	71.0 [67.0–75.5]	69.3 [64.5–73.4]	0.003 **
MBP (mmHg)	84.8 [82.2–88.9]	81.6 [76.0–85.8]	<0.001 ***
HR (bpm)	75.8 [69.9–80.0]	73.8 [68.4–79.0]	0.354
OPP (mmHg)	46.3 [44.1–49.1]	45.3 [42.0–47.7]	<0.001 ***
sAA (KU/L)	3.0 [3.0–9.8]	3.0 [3.0–3.0]	0.025 *
VAS (cm) eye strain	2.9 [1.8–5.3]	1.9 [0.8–3.8]	0.017 *
VAS (cm) stiff shoulder	3.6 [0.8–6.5]	0.9 [0.2–2.9]	<0.001 ***
mMBR	13.9 [12.1–17.8]	12.4 [10.6–15.7]	<0.001 ***
mMBR (%)	100 [100–100]	89.2 [84.7–94.2]	<0.001 ***
sMBR	185.5 [171.1–200.7]	169.1 [163.5–176.9]	0.005 **
sMBR (%)	100 [100–100]	93.3 [87.7–98.9]	0.009 **
Strain ratio	0.5 [0.4–0.5]	0.4 [0.2–0.5]	0.001 **

IOP, intraocular pressure; SBP, systolic blood pressure; DBP, diastolic blood pressure; MBP, mean blood pressure; HR, heart rate; OPP, ocular perfusion pressure; sAA, salivary α-amylase activity; mMBR, macular mean blur rate; sMBR, skin mean blur rate. Wilcoxon signed-rank test. Median [Q1–Q3]. * *p* < 0.05 ** *p* < 0.01 *** *p* < 0.001.

## Data Availability

The original contributions presented in this study are included in the article. Further inquiries can be directed to the corresponding author.

## Abbreviations

The following abbreviations are used in this manuscript:

DBP	Diastolic Blood Pressure
HR	Heart rate
IOP	Intraocular Pressure
LSFG	Laser Speckle Flowgraphy
MBP	Mean Blood Pressure
OPP	Ocular Perfusion Pressure
sAA	Salivary α-Amylase
SBP	Systolic blood pressure
VAS	Visual analog scale
BCVA	Best Corrected Visual Acuity
